# Metabolic alterations in dairy cattle with lameness revealed by untargeted metabolomics of dried milk spots using direct infusion-tandem mass spectrometry and the triangulation of multiple machine learning models†

**DOI:** 10.1039/d2an01520j

**Published:** 2022-10-26

**Authors:** Wenshi He, Ana S. Cardoso, Robert M. Hyde, Martin J. Green, David J. Scurr, Rian L. Griffiths, Laura V. Randall, Dong-Hyun Kim

**Affiliations:** a Centre for Analytical Bioscience, Advanced Materials & Healthcare Technologies Division, School of Pharmacy, University of Nottingham Nottingham NG7 2RD UK dong-hyun.kim@nottingham.ac.uk; b School of Veterinary Medicine and Science, University of Nottingham Sutton Bonington Campus Leicestershire LE12 5RD UK

## Abstract

Lameness is a major challenge in the dairy cattle industry in terms of animal welfare and economic implications. Better understanding of metabolic alteration associated with lameness could lead to early diagnosis and effective treatment, there-fore reducing its prevalence. To determine whether metabolic signatures associated with lameness could be discovered with untargeted metabolomics, we developed a novel workflow using direct infusion-tandem mass spectrometry to rapidly analyse (2 min per sample) dried milk spots (DMS) that were stored on commercially available Whatman® FTA® DMPK cards for a prolonged period (8 and 16 days). An orthogonal partial least squares-discriminant analysis (OPLS-DA) method validated by triangulation of multiple machine learning (ML) models and stability selection was employed to reliably identify important discriminative metabolites. With this approach, we were able to differentiate between lame and healthy cows based on a set of lipid molecules and several small metabolites. Among the discriminative molecules, we identified phosphatidylglycerol (PG 35:4) as the strongest and most sensitive lameness indicator based on stability selection. Overall, this untargeted metabolomics workflow is found to be a fast, robust, and discriminating method for determining lameness in DMS samples. The DMS cards can be potentially used as a convenient and cost-effective sample matrix for larger scale research and future routine screening for lameness.

## Abstract 2

Metabolic alteration and metabolite indicators associated with cattle lameness were discovered by untargeted metabolomics analysis of dried milk spots using direct infusion mass spectrometry and triangulation of statistical models.

## Graphical abstract

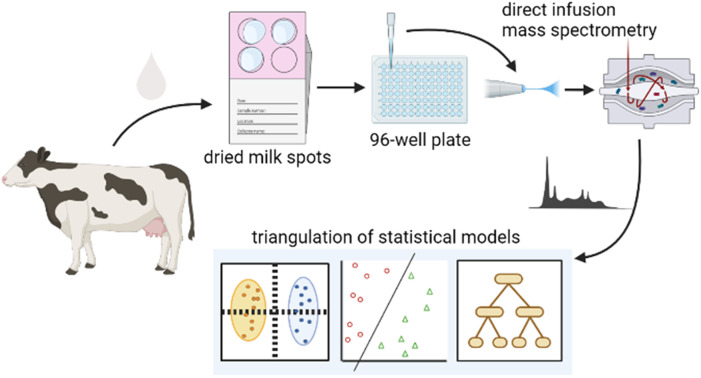

## Introduction

Lameness is a major health issue of dairy cows. It impairs sustainability due to animal health and welfare impacts, and therefore has economic and ethical implications.^1^ Despite the recent efforts by the dairy industry to reduce lameness levels, a recent study showed that the average prevalence in the UK is as high as 30.1%.^2,3^ Early detection and timely treatment are essential to mitigate the impacts of lameness.^4^ The current mainstream method of diagnosis is mobility scoring based on visual assessment of gait by trained observers.^5^ However, pain experienced by lame cows is often masked by their instinctive stoicism, which makes it difficult to diagnose the disease before the appearance of clinical signs.^6^ Another major limitation of this method is intra- and inter-observer variability.^7^ Other authors have reported that pro-inflammatory cytokines and acute-phase proteins (APPs) can be used as biomarkers.^8,9^ However, because of the high cost of ELISA tests required for detecting these immune-related molecules, alternative rapid and robust approaches are urgently required for routine screening.

Metabolomics has become an increasingly popular “omics” approach to biomarker discovery.^10^ State-of-the-art metabolomics techniques allow the detection of hundreds to thousands of metabolites with a minimal amount of sample.^11^ It is believed that metabolomics can deliver remarkable achievement in livestock research due to its capability of fast, effective and quantitative metabolic phenotyping.^12^ However, few studies have been reported regarding metabolic alteration associated with lameness. Zheng *et al.* utilised nuclear-magnetic-resonance (NMR)-based metabolomics to investigate metabolic difference between healthy and cows with footrot from blood samples.^13^ Dervishi *et al.* used gas chromatography-mass spectrometry (GC-MS) to investigate the metabolic signatures from serum samples of lame cows during different stages of lameness development.^14^ Eckel *et al.* showed that metabolic alterations during disease development could be identified from cow's urine using liquid chromatography (LC)-MS.^15^ However, metabolic alterations in lame cows have not yet been investigated using milk, which is a desired source as it is easily accessible and can be collected in a non-invasive manner.

In real-world settings, farmers may face logistical challenges sending samples from farms to laboratories for lameness diagnosis using metabolomics techniques. This arises from the need for temperature regulations (usually at −80 °C) during storage and transportation of conventional liquid biological samples. Hence, dried matrix (*i.e.*, blood, urine, milk) spots on paper is a more attractive sample type because of low sample volume required, ease of collection, room–temperature storage, and low–cost postal shipping. Although dried blood spots (DBSs) have been used in a wide range of research including metabolomics studies,^16^ few studies explored the use of different dried milk spots systems,^17–19^ and none for veterinary or agricultural applications. These established dried milk spot (DMS) systems studied human breast milk and often require pre-treatment of papers using different protocols which can introduce extra inconsistencies between studies. Here, we propose that the commercially available Whatman® FTA® DMPK cards, which is originally designed for DBSs, can be potentially used as a simpler way of collecting, preserving, and storing bovine milk samples for metabolomics research.

In metabolomics research, popular statistical approaches of identifying metabolic differences between classes are multivariate analysis techniques such as orthogonal partial least squares (OPLS) and univariate analysis (*e.g.* Student's *t*-test^20–23^). However, these “conventional” methods have innate limitations, especially when handling complex metabolomic data. Firstly, OPLS tends to construct prediction models that remove systematic variation that does not agree with the assigned group classification, therefore, force scores-space separation.^24^ Without rigorous validation, the “significant” results and “important” variables could be generated by the model solely by chance. Secondly, for Student's *t*-test, the idea of hypothesising “there is a difference” based on the concept of statistical significance and *p* values has been increasingly criticised, as it provides fairly limited information about the data, and can be easily misinterpreted.^25^ The triangulation of multiple machine learning methods can yield valuable insights on the reliability of the results generated from the statistical workflow described above. It can also mitigate the issue of results being method-dependent and improve the likelihood of identifying truly important variables.^26^ Furthermore, since covariate selection using conventional regression approaches often have high variability and relatively low reproducibility, stability selection could be incorporated into prediction models.^27–30^ This strategy can help identify the most stable predicators under resampling that are likely to be the strongest candidates as disease indicators among significant metabolites.

Here, we investigated the metabolic alterations in lame cows compared with non-lame cows and assess the suitability of Whatman® cards as a DMS media by using a direct infusion method with TriVersa NanoMate sampling system coupled to high-resolution MS. This direct infusion method allows high-speed analysis (2 min per sample) in ambient environment.^31^ This feature allows rapid screening for potential biomarkers which may also make it possible to conduct large-scale research and routine lameness testing for dairy cows in the future. Furthermore, with the strategy of using triangulation of multiple statistical models, we were able to identify potential disease predictors.

## Experimental

### DMS sample preparation

**Fig. 1:**
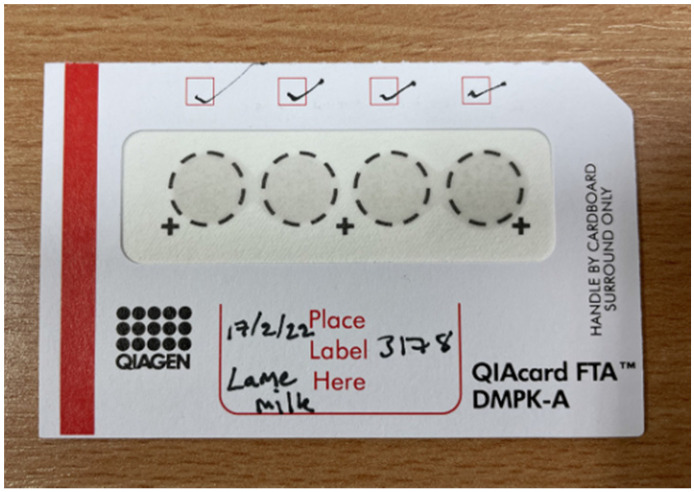
Example of dried milk spots on a Whatman® FTA® DMPK card.

Milk drops were collected directly onto Whatman® FTA® DMPK cards (Fig. 1) from 10 lame cows and 11 healthy cows from one dairy farm based at the Centre for Dairy Science Innovation (CDSI), University of Nottingham. It was a research dairy herd containing 300 cows that produce milk commercially. Cows were housed continuously with sand bedded cubicles and slatted flooring. Lame and healthy control cows were identified based on visual assessment using the Agriculture and Horticulture Development Board (AHDB) scoring system (0 to 3) where lame was defined as score ≥2 and healthy (non-lame) defined as score <2.^32^ Each spot on the FTA® DMPK cards contained one drop of milk (∼20 μL). The DMS cards were air-dried, then stored in plastic seal bags at room temperature. After 8 days, part of each spot was removed from the cards into 1.5 mL Eppendorf tubes (Eppendorf AG, Hamburg, Germany) using a 6 mm hole puncher. Extraction of metabolites from each sample was conducted with a 500 μL mixture of 70% v/v methanol (VWR, West Sussex, UK) and 30% v/v water to which MS-grade formic acid (Optima LC−MS grade; Fisher Scientific, Loughborough, UK) was added (final concentration, 0.1% v/v). Deionised water was prepared using a Milli-Q water purification system (Millipore, MA, USA). After mixing and incubating in the extraction solvent for 20 min, the samples were centrifuged (MiniSpin®, Eppendorf AG, Hamburg, Germany) for 10 min at 6708*g*. Then, 200 μL supernatants were transferred to clean Eppendorf tubes. To dilute the extracted metabolites, a further 800 μL extraction solvent was added to each sample. The procedure was adopted from a metabolite extraction method using dried blood spots by Trifonova *et al.*^33^ To assess the sample stability during a prolonged storage time at room temperature, the same metabolite extraction procedure was repeated on day 16 using adjacent milk spots.

### Mass spectrometry analysis

The solvents containing extracted metabolites were transferred into a 96-well plate, then 10 μL were directly infused into a high-resolution Q-Exactive plus Orbitrap spectrometer (Thermo Fisher Scientific, Hemel Hempstead, UK) *via* chip-based nanoelectrospray ionisation (Advion Biosciences, Ithaca, NY) at 1.5 kV and 0.6 psi gas pressure. Data was acquired for 1 min for each polarity using a scan range of *m*/*z* 70–1050. In full MS mode, the resolution was set to 140 000 at *m*/*z* 200, and the AGC target was set to 3 × 10^6^ with a maximum ion injection time of 200 ms. The top 10 most intense ions were isolated within a 0.5 *m*/*z* window for data-dependent acquisition (DDA) at a resolution of 17 500, AGC target of 1 × 10^6^ and a maximum ion injection time of 50 ms. For data-independent acquisition (DIA), the pre-selected ions were isolated within a 0.4 *m*/*z* window at a resolution of 35 000 and AGC target of 2 × 10^5^. Stepped normalized collision energy (NCE) of 20, 30 and 40 was applied in both DDA and DIA. The pooled QC samples were analysed intermittently for the duration of the MS analysis.

### Peak picking and alignment

The .RAW data files from Xcalibur were converted to .mzXML format using ProteoWizard.^34^ Peaks with intensities above 100 000 were picked and aligned within a 5 ppm *m*/*z* window using an in-house MATLAB (R2020a, The MathWorks, Inc., Natick, MA) script.^11^ Features with more than 20% missing values across all samples were removed. The remaining missing values were imputed using *k*-nearest neighbour (*k*nn) imputation (*k* = 10).^35^ Individual ion intensity matrices from both polarities were concatenated using a low-level data fusion strategy.^36^

### Multivariate and univariate analysis

Following the feature extraction workflow, the data were normalised to total ion count, log-transformed and Pareto scaled.^37^ Principal component analysis (PCA) and orthogonal partial least squares discriminant analysis (OPLS-DA) models were constructed using SIMCA 16 software (Umetrics, Sweden). Selection of discriminative variables was based on a variable's importance in projection (VIP) score > 1 in OPLS-DA models. The models were validated using the built-in function of leave-one-out cross validation (LOOCV) procedure and permutation test. Student's *t*-test with controlled false discovery rate (FDR) (*q* < 0.05) was performed in MetaboAnalyst 5.0.^38,39^ A *p*-value < 0.05 was considered significant.

### Machine learning and stability selection

Following the feature extraction workflow, the data were normalised to total ion count and standardised. Four common supervised machine learning (ML) techniques were performed in R,^40^ including random forest (RF),^41^ elastic net,^42^ partial least squares (PLS),^43^ and support vector machine (SVM).^44^ Prediction accuracy of each model was assessed using LOOCV: for each ML algorithm, 1 cow was chosen as test set and the remaining cows were used as training set. This procedure was repeated 20 times for each model. Recursive feature elimination (RFE) was used for all algorithms to identify the smallest set of metabolites that provided maximum predictive accuracy; this was conducted external to the LOOCV procedure to ensure no selection bias occurred.^45^

Stability selection was performed using the stabiliser package.^46^ Three penalised models: elastic net,^42^ minimax convex penalty (MCP)^47^ and least absolute shrinkage and selection operator regression (Lasso)^48^ were constructed. Selection stability was evaluated for each model as the percentage of times that each variable was selected across 500 bootstrap samples.^49^ To estimate the stability threshold, the outcome was permuted 20 times to generate 20 new datasets in which the relationship between the outcome and observations were severed. The threshold was determined by the highest stability score achieved in the permutated datasets over 50 bootstrap samples across each of the 20 permuted datasets.^50^ A bootstrap *p*-value was defined as the proportion of coefficient estimates on the minority side of zero. For example, if a variable was selected on 100 occasions and the coefficients on 95 occasions were greater than 0, then the bootstrap *p*-value would be (100 − 95)/100 = 0.05.

### Metabolite identification

The ion masses of important variables were searched against the Bovine Metabolome Database^51^ with 5 ppm mass tolerance and Lipid Maps^52^ with ±0.001 *m*/*z* tolerance using [M + H]^+^, [M + Na]^+^, [M + K]^+^, and [M + H − H_2_O]^+^ as adducts for positive mode, also [M – H]^−^ and [M − H − H_2_O]^−^ for negative mode. For lipid search, multiply charged adducts were also included. For structure-based identification, MS/MS spectra were matched with the experimental reference spectra from the same normalised collision energy using mzCloud by comparing the fragmentation patterns and the accurate mass of the fragments. For compounds that were not recorded in mzCloud database, accurately predict ESI-MS/MS spectra generated by CFM-ID program were used for improving confidence for identification.^53^

## Results and discussion

### Rapid metabolic profiling workflow for the investigation of dairy cow lameness

**Fig. 2:**
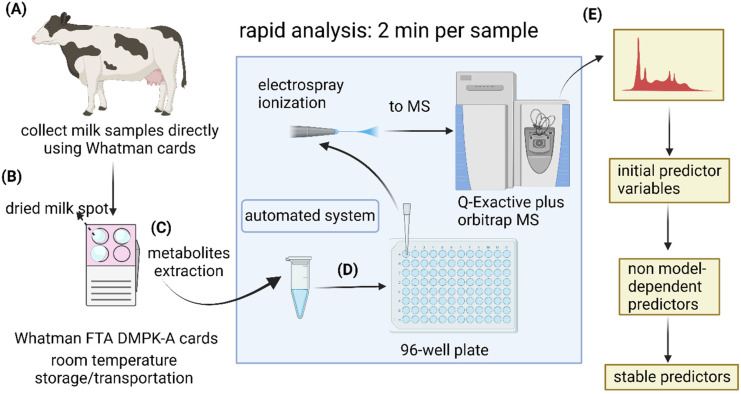
Rapid analysis of dried milk spot cards with direct infusion mass spectrometry. (A) Milk drops were collected directly from cows onto commercially available Whatman FTA® DMPK Cards. (B) Milk spots were air-dried, then stored in plastic seal bags at room temperature for 8 and 16 days. (C) Spot cards were punched for metabolite extraction. (D) The extraction solvent containing milk metabolites was transferred into a 96-well plate, then delivered to mass spectrometer using the automated sampling system, TriVersa NanoMate LESA®. (E) Multivariate and univariate analysis were first carried out for class predication and identification of lameness-related metabolites. The results were further validated using machine learning and stability approach.

Commercially available Whatman FTA® DMPK cards were used to collect milk drops directly from dairy cows (Fig. 2A and B). To evaluate analytes stability during prolonged storage periods, the dried milk spot (DMS) cards were stored in plastic seal bags at room temperature for 8 and 16 days, respectively, until metabolite extraction (Fig. 2C). The extracted samples were directly infused into a high-resolution mass spectrometer for rapid metabolomic analyses (2 min per sample) using a robotic sampling system (Fig. 2D). The idea behind this workflow was to explore the use of DMS sample matrix for easy sample collection and low-cost postal delivery from farms to analytical laboratories for rapid lameness diagnosis. This could potentially be an attractive option as it omits the needs for temperature regulations during storage and transportation for conventional liquid samples. It also eliminates the inter- and intra-person variability in comparison to the current diagnostic approach. For laboratories, the simple sample preparation procedure and rapid analysis with direct infusion system could allow high throughput for large-scale veterinary clinical research. For data analysis, we added machine learning and stability test to the conventional workflow of OPLS-DA and *t*- test to mitigate the issue of results being method-dependent and identify the most stable predictors (Fig. 2E)

### Metabolic profiles of DMS differentiate lame and control cows

**Fig. 3:**
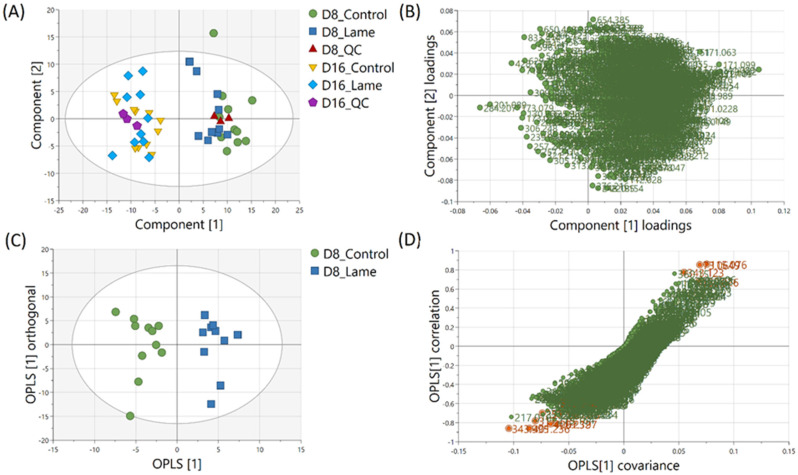
Multivariate analysis results. (A) PCA of dried milk spots extracted on day 8 and day 16 after sample collection. Pooled QC samples showed stable analytical performance. (B) Loadings of PCA principal component 1 and 2. (C) OPLS-DA scores plot reveals clustering of cows based on health conditions (control *vs.* lame) (*R*^2^*X* 0.321 *R*^2^*Y* 0.899 *Q*^2^ 0.601) from milk metabolites extracted on day 8. (D) S plot shows ions that have strong correlation with the cow health conditions (orange).

The milk metabolic profiles acquired by direct infusion MS were used to discriminate the four sample groups (day 8 extracts – control/lame, day 16 extracts – control/lame). Features in positive and negative ion modes were combined and used to construct a PCA model (number of components *A* = 6, number of observations *N* = 48) (Fig. 3A),^54^ in which all DMS extracted on day 8 and day 16 since sample collection are clearly distinguished in the first principal component (PC 1) (*x*-axis). In the PCA plot, the pooled QC samples located in the middle of all analysed samples from the same extraction day, which indicated good reproducibility during the analytical run. The PC 1 loadings plot revealed an overall reduction in signals from day 16 samples compared to day 8 (Fig. 3B).

An OPLS-DA model was built (*A* = 1 predictive component +1 orthogonal component, *N* = 21) to compare the healthy group and lame group from day 8 extracted metabolites (Fig. 3C). The model was validated using LOOCV method. Clear grouping of the two classes was observed (*Q*^2^: 0.601). In general, *Q*^2^ (goodness-of-prediction) > 0.5 is considered as good predictability,^55^ and 0.4 may also be considered acceptable for biological models.^11,23^ To mitigate the issues with potential overfitting and over estimation of Q^2^, we further conducted a permutation test which confirmed the validity of the constructed model (Fig. S1†). The associated S-plot enabled the determination of the most important ions for distinguishing the control and lame cows (Fig. 3D).^56^ The discriminative ions (highlighted in orange colour) were determined by a VIP score > 1 in OPLS-DA and a *p* value < 0.05 in multiple *t*-test (FDR corrected, *q* < 0.05). Ten out of 12 discriminative ions were assigned putative molecular formulae (Table 1). To further confirm the identities of these discriminative ions, both experimental and computed MS/MS spectra were used for structure-based identification (Fig. S3–S8†).

**Table 1:** Annotation for discriminative ions (VIP > 1, *p*-value < 0.05, FDR corrected) of healthy and lame cow groups (day 8). VIP: variable importance in the projection. FDR: false discovery rate

*m*/*z*	Adduct	Assignment	Mass error/ppm	Monoisotopic mass (Da)	Identification method
343.995	Unknown	Unknown	Unknown	Unknown	Unknown
315.0416	Unknown	Unknown	Unknown	Unknown	Unknown
267.1968	[M − H_2_O − H]^−^	Hexadecanedioic acid	3.0	286.2144	*m*/*z*
401.2358	[M + 2Na]^2+^	PG 35:4	1.2	400.2283	*m*/*z*, computed MS/MS
317.1149	[M + K]^+^	Alpha-carboxyethyl hydrochroman	0.3	278.1518	*m*/*z*
115.0757	[M + H − H_2_O]^+^	6-Hydroxyhexanoic acid	−1.7	132.0786	*m*/*z*, MS/MS
251.1408	[M + K]^+^	Trans-11-methyl-2-dodecenoic acid	0.0	212.1776	*m*/*z*, MS/MS
166.0258	[M + K]^+^	1-Piperideine-2-carboxylic acid	−4.2	127.0633	*m*/*z*, computed MS/MS
73.0649	[M + H]^+^	Isobutylaldehyde	1.4	72.0575	*m*/*z*, MS/MS
400.2321	[M + H]^+^	Carnitine 13:3;O3	2.2	399.2257	*m*/*z*
202.0685	[M + Na]^+^	Glucosamine	−0.5	179.0793	*m*/*z*
343.1228	[M + H]^+^	Alpha-Lactose	−2.0	342.1162	*m*/*z*, MS/MS

### Triangulation of machine learning models for results validation

**Fig. 4:**
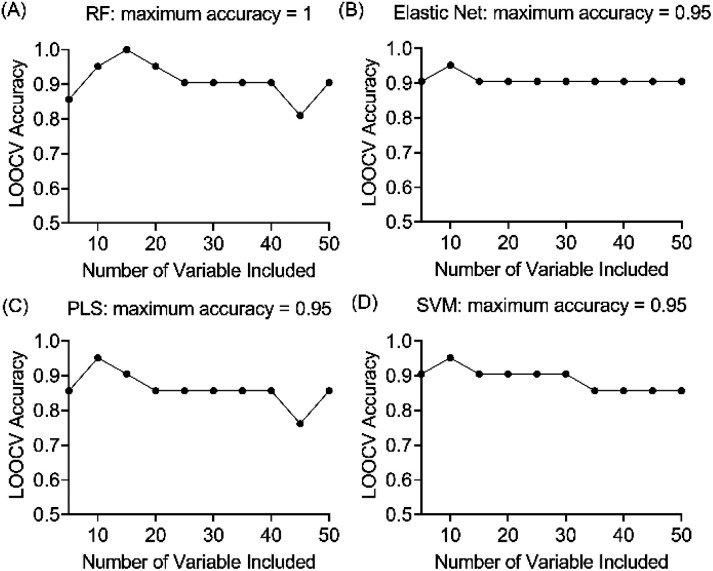
Evaluation of the prediction accuracies of four ML models (A) random forest (RF), (B) elastic net, (C) support vector machine (SVM), (D) partial least squares (PLS) using leave-one-out cross validation procedure.

**Fig. 5:**
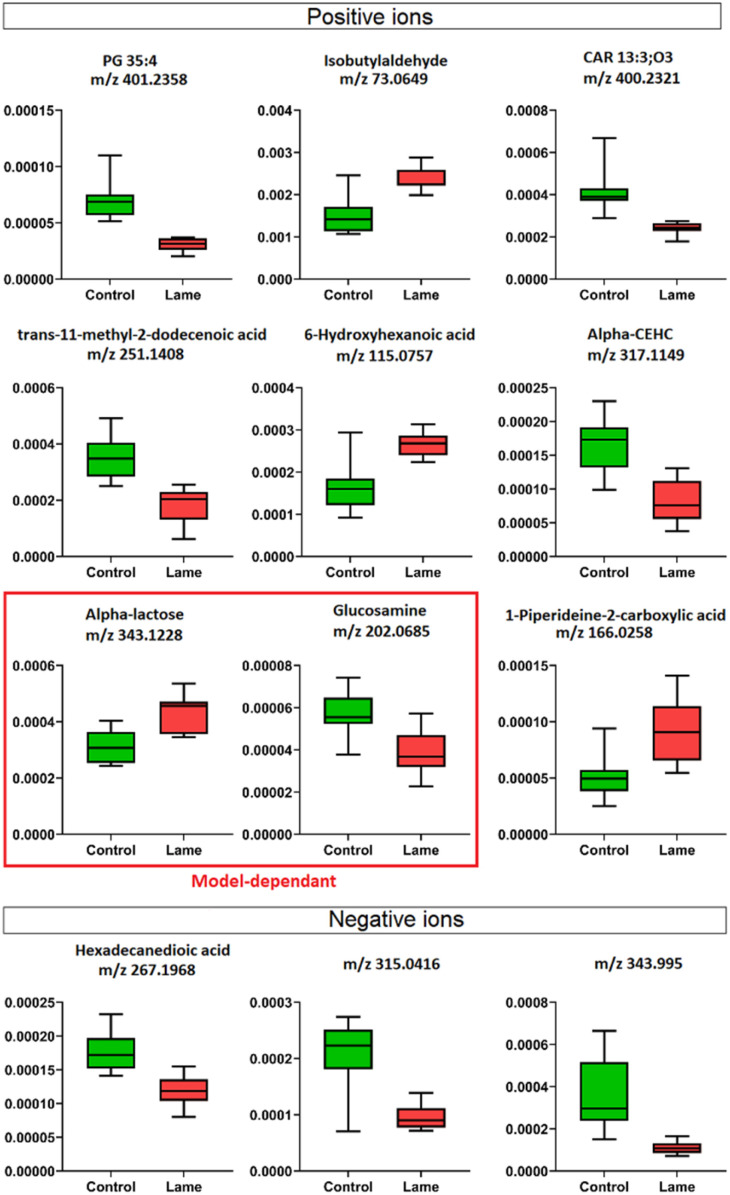
Box plots show the relative abundance of discriminative metabolites from day 8 between healthy and lame cows determined by OPLS-DA and Student's *t*-test (VIP score > 1, *p* < 0.05). Validating the results by triangulation of multiple machine learning models, we identified two model-dependent predictors, alpha-lactose and glucosamine, which were chosen as “important” predictor only in PLS-based methods, therefore, not likely to be “true” predictors.

Four machine learning models: RF, elastic net, PLS, and SVM were tested by recursive feature elimination and LOOCV (Fig. 4). In RF, a maximum predictive accuracy of 100% was achieved with 15 selected variables. Elastic net, PLS and SVM reached the highest accuracies of 95.2% with 10 selected variables (Table S1†). Comparing the top 10 most important variables (Table S2†) selected by each ML model with the discriminative ions from the conventional workflow based on OPLS and *t*-test (Table 1), we observed high similarities between variable selections. Interestingly, results from PLS and OPLS methods were in full agreement, which is probably because the models are constructed based on similar concepts.^55^ From the 12 discriminative metabolites discovered in the conventional workflow, *m*/*z* 202.0685 (glucosamine) and *m*/*z* 343.1228 (alpha-lactose) were selected only when (O)PLS was applied (*i.e.*, model-dependent). Therefore, it is likely that they may not truly associate with the disease state. The remaining 10 metabolites were selected as predictor variables in multiple distinct models (Fig. 5).

Lipids are important metabolic fuel, and they have various functions in cell activation, immune response and inflammation.^14^ In this study, a few fatty acids in milk were discovered to play an important role in discriminating the lame and healthy cows. From lame cows, a relative decrease was observed in a saturated long-chain fatty acid (hexadecanedioic acid) and an unsaturated fatty acid (*trans*-11-methyl-2-dodecenoic acid) compared with healthy cows. In contrast, the lame cows had a relatively elevated abundance in an omega-hydroxy fatty acid called 6-hydroxyhexanoic acid. Other significantly altered lipids were phosphatidylglycerol PG 35:4, and fatty acyl carnitine CAR 13:3;O3, which both had a decrease in the lame group compared to the healthy group. In previously reported studies using plasma and urine samples, distinct metabolite profiles between lame cows and controls were displayed by a number of acylcarnitines and glycerophospholipids.^15,57^ The alteration in these lipid species were linked to inflammation and immune response. For acylcarnitines, they also play an important role in the lipid β-oxidation process.^57^

Interestingly, while most reported lipid markers in serum or plasma displayed elevated concentration in lame cows, we discovered many lipid predictors with decreased abundance in milk in this study. Further investigation is required to determine the underlying reasons for these alterations in milk. In addition, we observed an increase in 1-piperideine-2-carboxylic acid, which is a metabolite in the pipecolic acid pathway of lysine degradation.^58^ In dairy cows, lysine is important for milk protein synthesis, carnitine synthesis, weight gain in growing cattle, and incorporation into mammalian tissues for structural integrity.^59^ The increased 1-piperideine-2-carboxylic acid in milk may indicate abnormal lysine metabolism in lame cows. Another significantly increased small molecule in lame cows is isobutylaldehyde. Its role in bovine metabolism is not yet fully elucidated.

### Selection of the most stable predictors

**Fig. 6:**
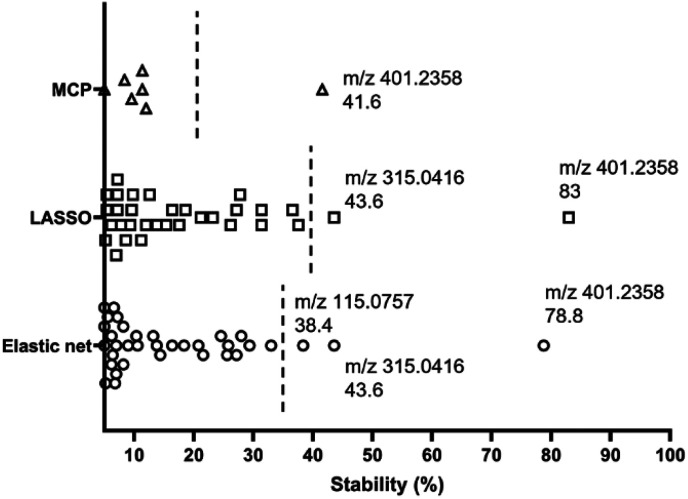
Variable selection and importance were visualised by plotting selection stability in elastic net, Lasso and minimax convex penalty (MCP) models. Variables that were never selected in any bootstrap or with a stability score below 5% were not shown in the figure. The line on the graph represents the calculated threshold to determine a cut-off for ‘important’ covariates.

High variability of results and low reproducibility is a common issue with conventional regression methods (*i.e.*, a single, non-bootstrapped regression model) for covariate selection from high dimensional data in comparison to stability selection.^27–30^ In our study, a majority of discriminative metabolites discovered in single machine learning models were not repeatedly selected during bootstrap resampling followed by one-off regression analyses indicated by their low stability scores (Table S3†). Bootstrap resampling is a statistical test that uses random sampling with replacement to mimic real-world sampling process. For instance, isobutylaldehyde was only selected as a “discriminative” metabolite in 9.6% to 36.6% resamples depending on the model types, which means it is highly likely that this metabolite will not be identified as a potential marker in another study where a single one-off regression model is employed. This can make biomarker screening challenging because the selected predictors may be incomparable between studies or analyses and fail to represent the target population. A solution to this issue is stability selection. The concept is that the variables truly associated with the outcome of interest are likely to be selected most frequently during multiple bootstrap resampling.^60^ Here, three penalised models elastic net, MCP and Lasso were implemented for selecting the most stable predictor metabolites.^61^ Selection stability was estimated for all models using a bootstrap methodology (500 bootstraps, 20 permutations, 50 permutation bootstraps).^30^ In each model, variables with a stability score above the estimated threshold and a low bootstrap *p*-value were selected (Fig. 6). The stability selected variables were *m*/*z* 401.2358 (PG 35:4), *m*/*z* 315.0416 and *m*/*z* 115.0757 (6-hydroxyhexanoic acid, C_6_H_12_O_3_) using the default elastic net model, which have also been selected using the triangulation method of OPLS-DA and ML as discussed in the previous section. It is noteworthy that *m*/*z* 401.2358 (PG 35:4) not only showed the highest stability scores in three stability models, but also had the highest importance rankings (top 2) in all ML models. Therefore, it appears to be the strongest candidate as an indicator of disease state. A Receiver Operator Characteristic (ROC) curve analysis was employed to further assess both the sensitivity and specificity performance of the predictor metabolites (Fig. S9†). Metabolite *m*/*z* 401.2358 (PG 35:4) showed a superior prediction performance with a sensitivity and specificity of 100%.

## Conclusions

A novel analytical workflow for untargeted metabolomics of dried milk spots (DMS) using direct infusion mass spectrometry was developed and it is shown to be a robust and discriminating approach for diagnosing lameness in dairy cows. Some important predictor metabolites have been discovered for the first time using the triangulation method of multiple statistical models including OPLS-DA, ML models and stability selection. This statistical workflow allowed identification of the most promising candidates for indicating lameness and eliminating model-dependent “predictors”, which vastly increased the reliability of the outcome. Phosphatidylglycerol and fatty acid species were found to be strong and sensitive candidates as indicators of lameness. Furthermore, we showed that Whatman® FTA® DMPK paper cards, a new sample media for milk collection, can be used for cost-effective and fast veterinary screening because it omits the need for temperature regulation often required by conventional liquid samples transportation and storage. DMS samples from healthy and lame cows can be clearly distinguished by their metabolite profiles after storing at room temperature for up to 8 days. This opens new opportunities to perform large-scale routine diagnosis for lameness, using milk as a sample that farmers can easily collect at low cost.

This experiment is a proof-of-concept study exploring the use of DMS as sample matrix for studying lameness by using untargeted metabolomics method. We acknowledge that the number of lame cows included in this study was low, and all cows were from the same farm. Future work should include larger cohorts of animals from multiple farms to further validate the current findings and determine the underlying reasons for observed metabolic alterations. Furthermore, future work should include the study of DMS samples from pre-lame cows, to determine whether this workflow can be used to predict lameness, and diagnose earlier than the current method which relies on the physical signs of lameness being apparent. This could then pave the way for early interventions in the future.

This developed analytical workflow and statistical strategy can also be applied to explore a wide range of diseases using dried liquid samples such as milk, blood and urine as a fast and robust untargeted method to determine the presence of potential biomarkers in the sample of choice.

## Conflicts of interest

There are no conflicts to declare.

## Supplementary Material

AN-147-D2AN01520J-s001
