# Supplementary material for: Metabolic alterations in dairy cattle with lameness revealed by untargeted metabolomics of dried milk spots using direct infusion-tandem mass spectrometry and the triangulation of multiple machine learning models
Source: Analyst. 2022 Oct 26;147(23):5537–45. doi: 10.1039/d2an01520j (PMC9678129; doi:10.1039/d2an01520j)
Supplement: AN-147-D2AN01520J-s001 [file AN-147-D2AN01520J-s001.pdf]

Electronic Supplementary Material (ESI) for Analyst.
This journal is © The Royal Society of Chemistry 2022

Supporting information for

Metabolic alterations in dairy cattle with lameness revealed by untargeted metabolomics of dried milk spots using direct infusion-tandem mass spectrometry and the triangulation of multiple machine learning models

Wenshi He,[a] Ana S. Cardoso,[b] Robert M. Hyde,[b] Martin J. Green,[b] David J. Scurr,[a] Rian L. Griffiths,[a] Laura V. Randall,*[b] and Dong-Hyun Kim*[a]

[a] Centre for Analytical Bioscience, Advanced Materials & Healthcare Technologies Division, School of Pharmacy, University of Nottingham, Nottingham NG7 2RD, U.K.

[b] School of Veterinary Medicine and Science, University of Nottingham, Sutton Bonington Campus, Leicestershire, LE12 5RD, U.K.

*Correspondence to: dong-hyun.kim@nottingham.ac.uk & laura.randall@nottingham.ac.uk

Supporting information

(**Fig. S1**) Permutation tests were performed on OPLS-DA models for validation

(**Fig. S2**) Comparison between dried milk spots (DMSs) extracted on day 8 and day 16 after sample collection

(**Table S1**) Prediction accuracies of four machine learning models when different numbers of variables included

(**Table S2**) Important variables selected using each machine learning model ranked from 1 to 10

(**Fig. S3-S6**) Metabolite identification based on MS2 using mzCloud database

(**Fig. S7**) Annotations of unsaturated hydrocarbons and glycerolipid backbone fragments of lipid phosphatidylglycerol (PG 35:4) (m/z 401.2358) in MS2 spectra

(**Fig. S8**) Annotations of cyclic fragments of 1-piperideine-2-carboxylic acid (m/z 166.0258) in MS2 spectra

(**Table S3**) Variable stability, coefficient estimates, 90 % boot-strap confidence intervals and bootstrap p-value for discriminative variables.

(**Fig. S9**) Receiver Operating Characteristic (ROC) Curves for the discriminative ions identified from the conventional OPLS-DA based statistical workflow.

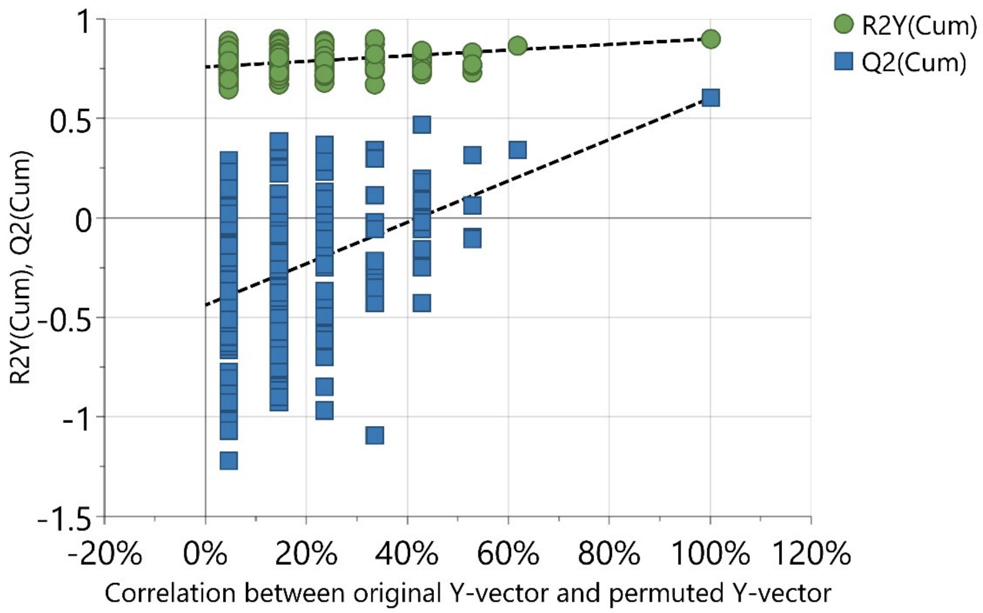

Fig. S1 Permutation tests were performed on OPLS-DA models for statistical validation. The permutation plot based on 200 random permutations indicates that the OPLS-DA model of day 8 metabolite extracts is valid as Q^2 values (blue squares) from the permuted test (bottom left) are lower than the corresponding original points (top right).

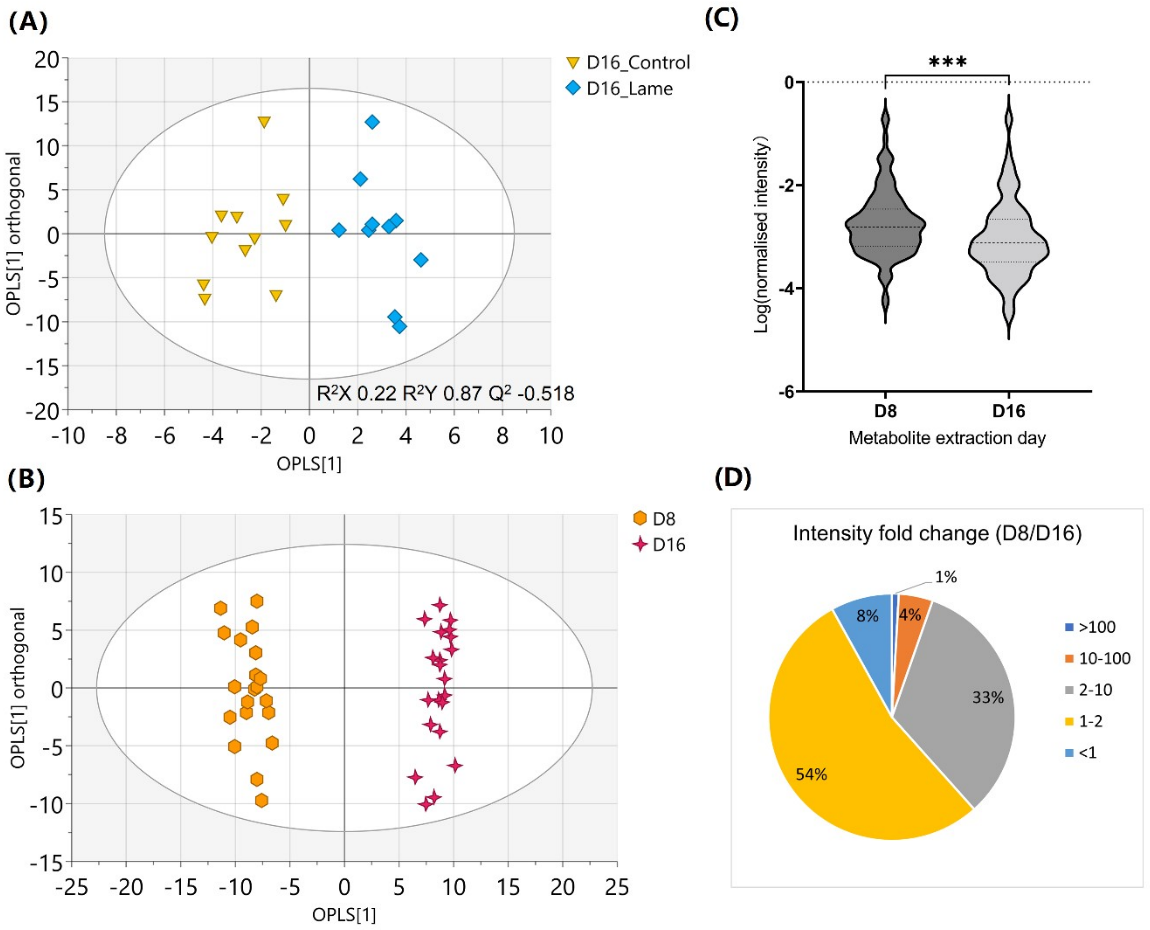

Fig. S2 Comparison between dried milk spots extracted on day 8 and 16 after sample collection. (A) OPLS-DA model of day 16 extracted metabolites shows low predictability (R^2X 0.22 R^2Y 0.87 Q^2 -0.518). (B) OPLS-DA model shows clear clustering between day 8 and day 16 extracted metabolites. A total 112 discriminant ions were discovered. (C) The violin plot reveals that discriminant ions from day 16 show overall reduced intensities compared to those from day 8. (D) The pie chart shows fold changes (day 8/day 16) of discriminant ions. Higher abundance was observed in a majority of 92% ions on day 8, indicating that the clustering between day 8 and day 16 samples was likely due to metabolites degradation with prolonged storage time.

Table S1 Prediction accuracies of four machine learning models when different numbers of variables included.

# Variables included	Mean repeat accuracy			
	Random forest	Elastic net	Support vector machine	Partial least squares
5	0.857	0.905	0.905	0.857
10	0.952	0.952	0.952	0.952
15	1.000	0.905	0.905	0.905
20	0.952	0.905	0.905	0.857
25	0.905	0.905	0.905	0.857
30	0.905	0.905	0.905	0.857
35	0.905	0.905	0.857	0.857
40	0.905	0.905	0.857	0.857
45	0.810	0.905	0.857	0.762
50	0.905	0.905	0.857	0.857

Table S2 Important variables selected using each machine learning models ranked from 1 to 10.

Rank	Random forest	Elastic net	Support vector	Partial least squares
1	400.2321	401.2358	400.2321	401.2358
2	401.2358	251.1408	401.2358	73.0649
3	343.995	400.2321	343.995	251.1408
4	73.0649	166.9863	251.1408	400.2321
5	251.1408	130.9651	267.1968	115.0757
6	115.0757	315.0416	317.1149	317.1149
7	267.1968	317.1149	73.0649	267.1968
8	553.3774	507.3997	217.0161	315.0416
9	317.1149	267.1968	105.0193	343.1228
10	217.0161	166.0258	115.0757	202.0685

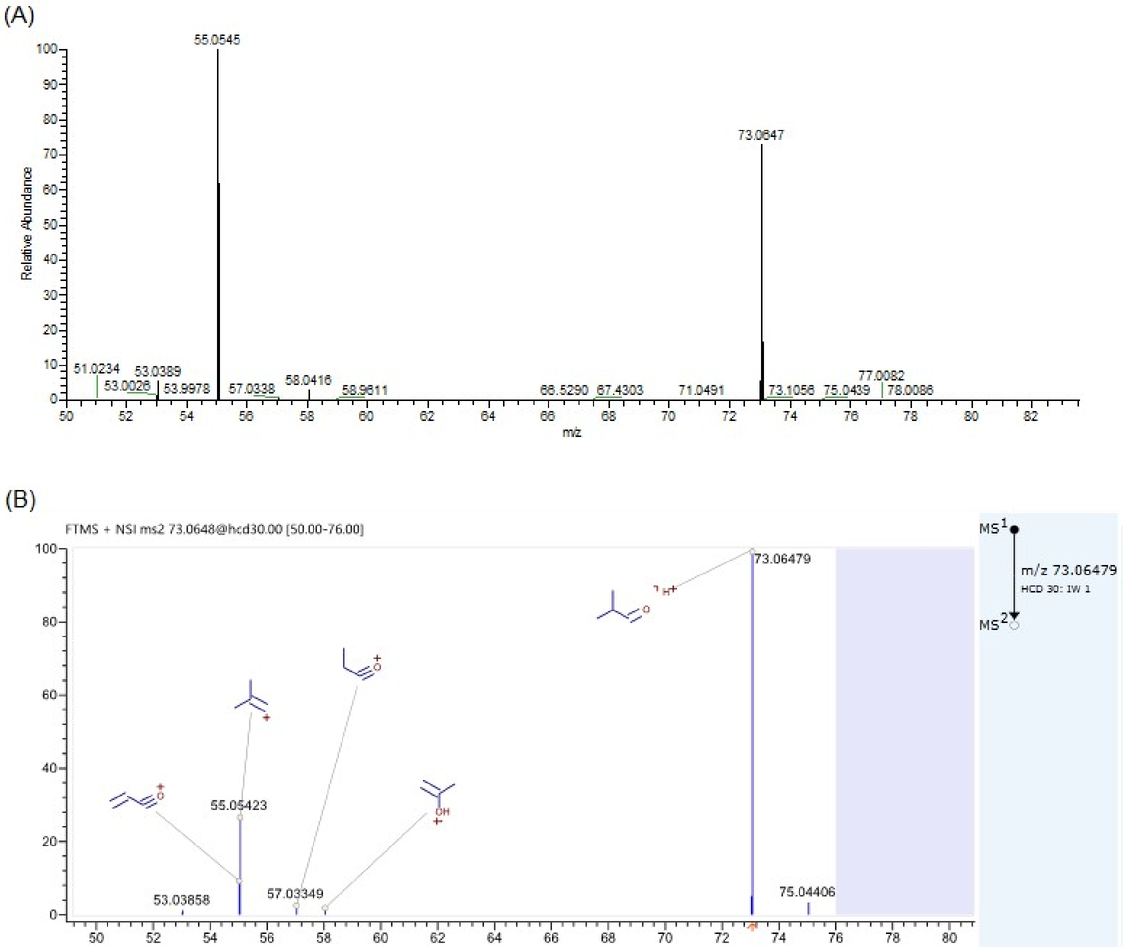

Fig. S3 Identification of isobutylaldehyde based on MS2 using mzCloud database. (A) Experimental MS/MS and (B) mzCloud reference spectra of m/z 73.0649 (isobutylaldehyde) with normalised collision energy HCD 30.

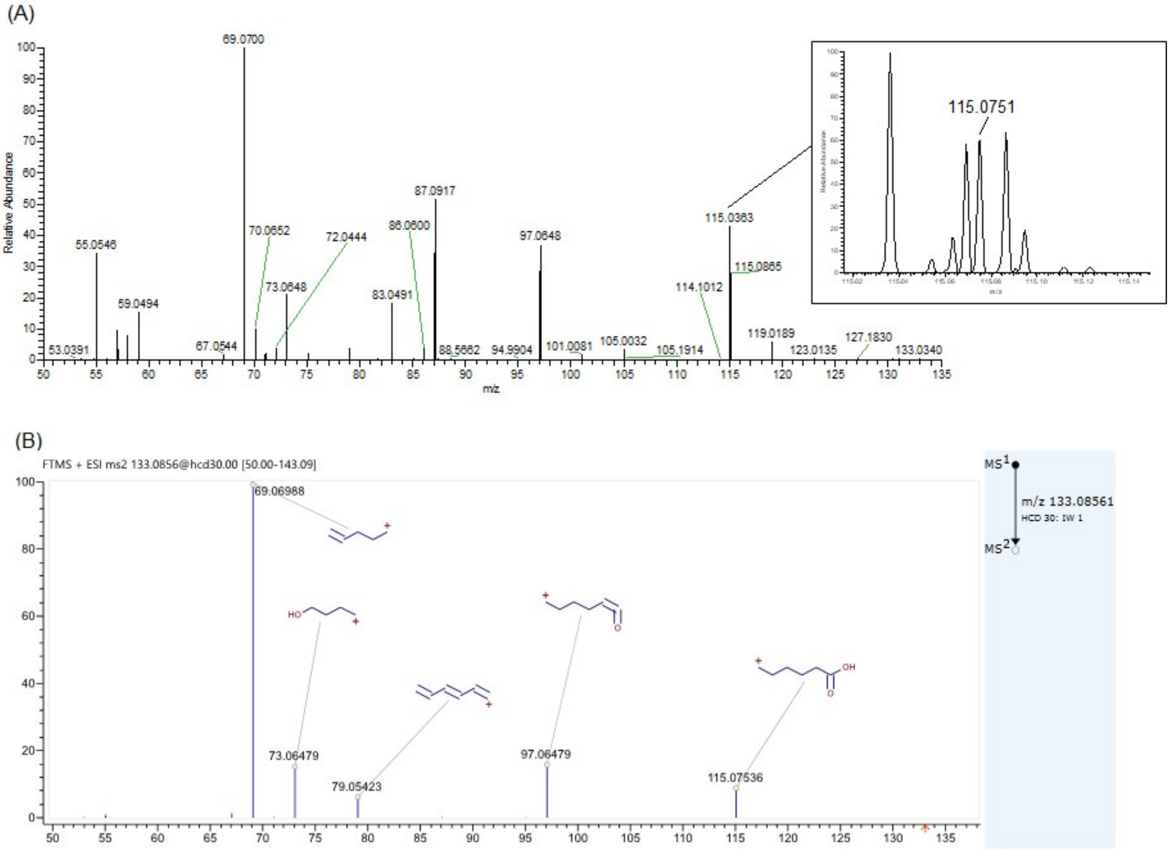

Fig. S4 Identification of 6-hydroxyhexanoic acid based on MS2 using mzCloud database. (A) Experimental MS/MS and (B) mzCloud reference spectra of m/z 115.0757 (6-hydroxyhexanoic acid) with normalised collision energy HCD 30.

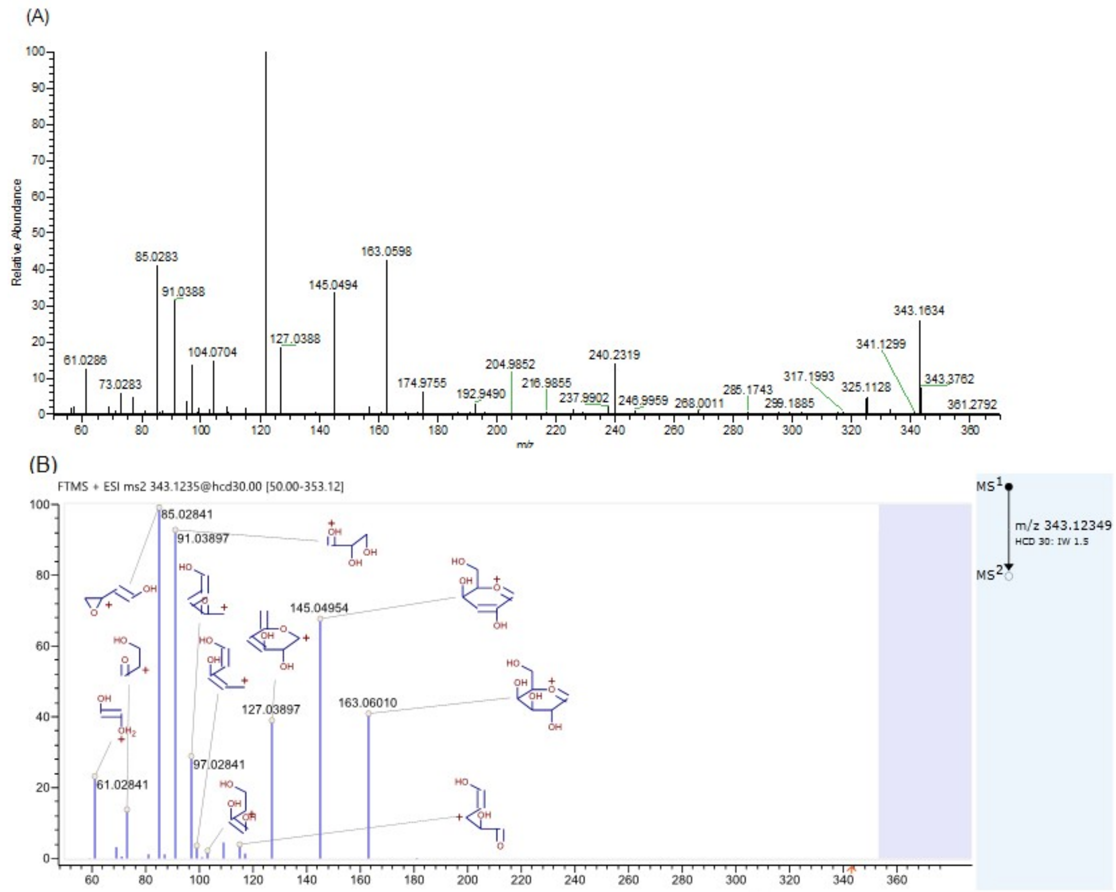

Fig. S5 Identification of alpha-lactose based on MS2 using mzCloud database. (A) Experimental MS/MS and (B) mzCloud reference spectra of m/z 343.1228 (alpha-lactose) with normalised collision energy HCD 30.

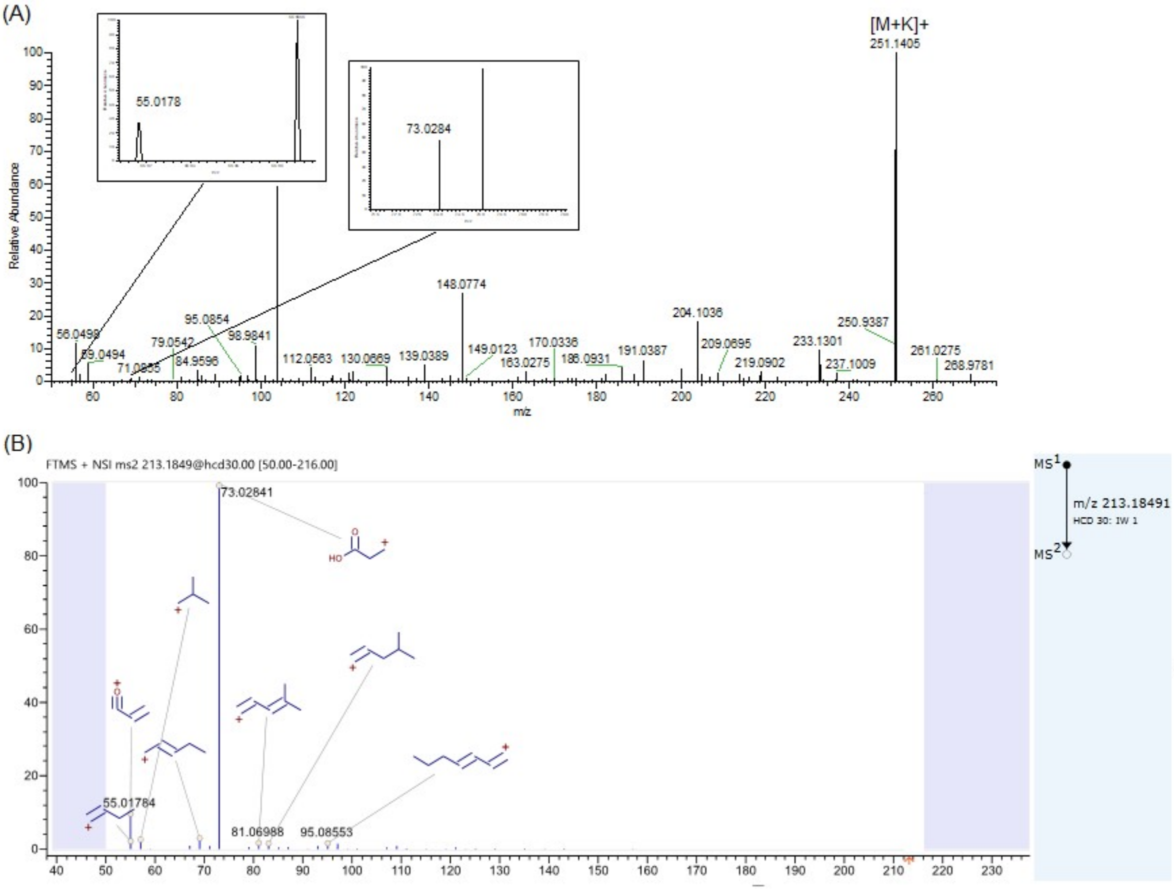

Fig. S6 Identification of trans-11-methyl-2-dodecenoic acid based on MS2 using mzCloud database. (A) Experimental MS/MS and (B) mzCloud reference spectra of m/z 343.1228 (trans-11-methyl-2-dodecenoic acid) with normalised collision energy HCD 30.

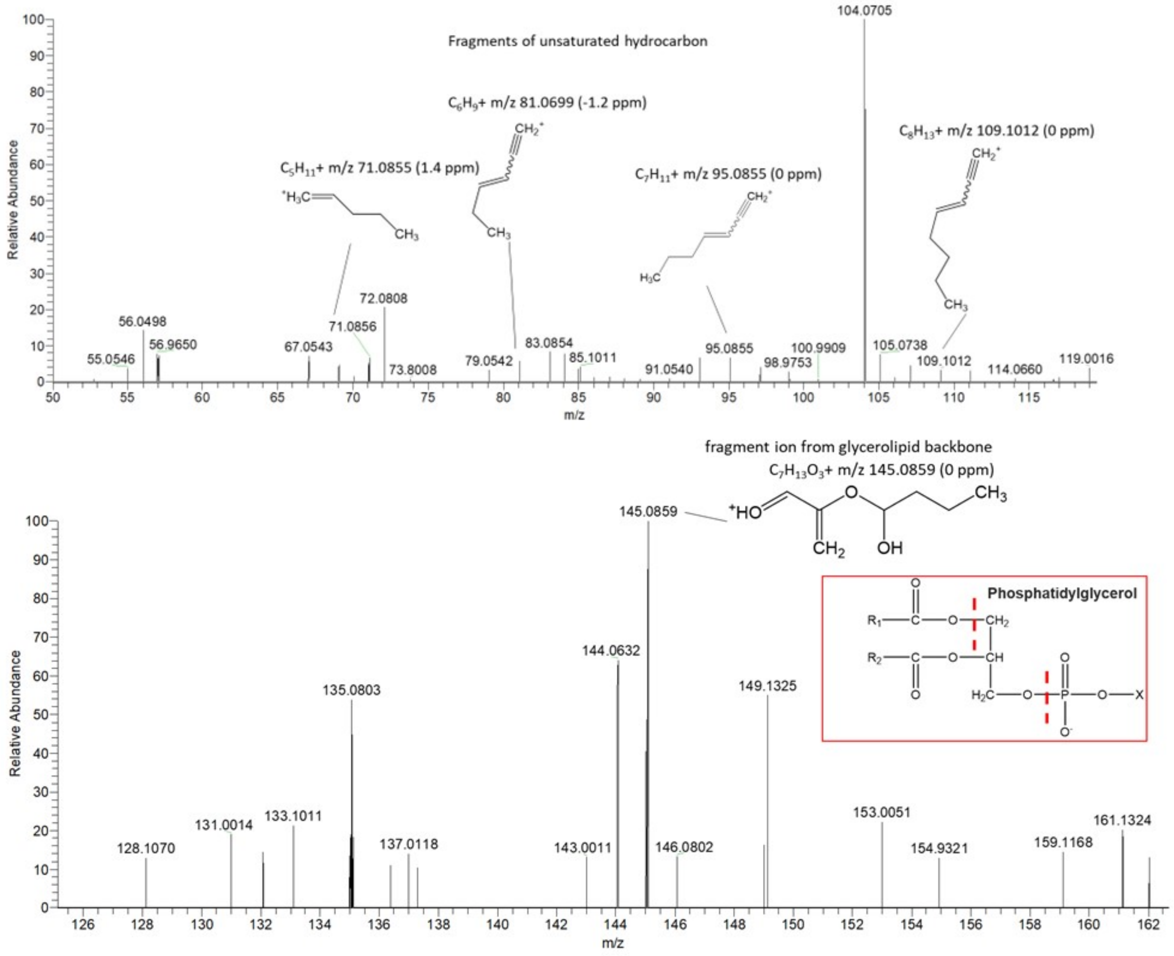

Fig. S7 Annotations of unsaturated hydrocarbons and glycerolipid backbone fragments of lipid phosphatidylglycerol (PG 35:4) (m/z 401.2358) in MS/MS spectra.

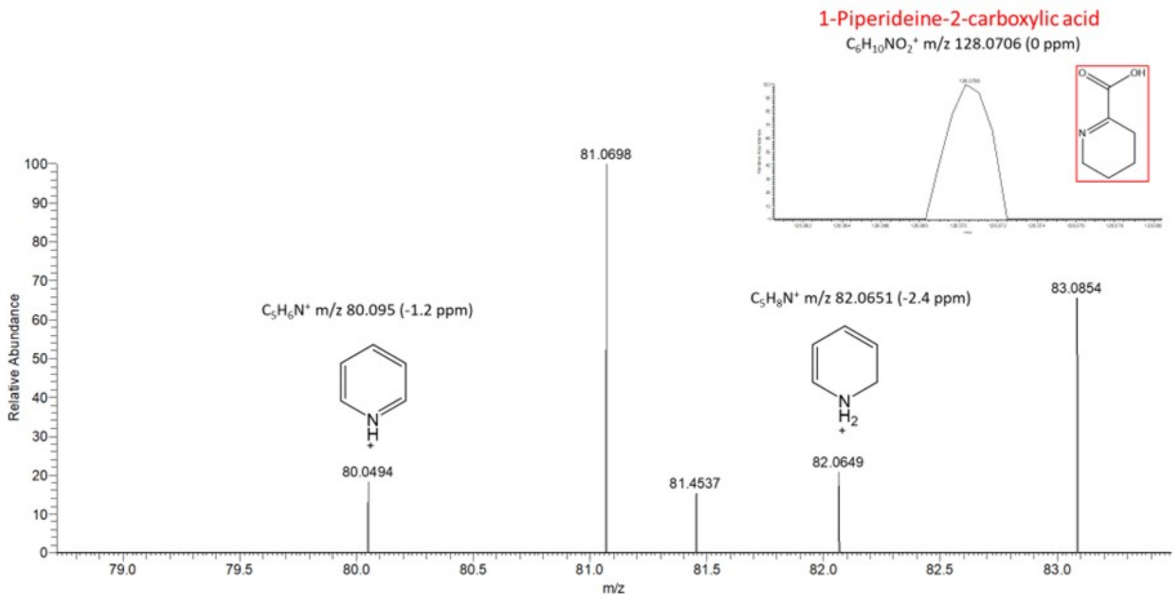

Fig. S8 Annotations of cyclic fragments of 1-piperideine-2-carboxylic acid (m/z 166.0258) in MS2 spectra.

Table S3 Variable stability, coefficient estimates, 90 % bootstrap confidence intervals and bootstrap *p*-value for discriminative variables. Elastic net, Lasso and minimax convex penalty (MCP) were applied for variable selection. Stability scores above the threshold were displayed in bold. VIP: variable importance in projection. OPLS-DA: orthogonal partial least squares discriminant analysis. FDR: false discovery rate. CI: confidence interval.

**Selected variables (OPLS-DA)**			**T test**		**Elastic net**					**Lasso**					**MCP**				
m/z	Polarity	VIP score	*p*-value	FDR	mean coefficient	lower CI	upper CI	bootstrap *p*-value	stability	mean coefficient	lower CI	upper CI	bootstrap *p*-value	stability	mean coefficient	lower CI	upper CI	bootstrap *p*-value	stability
343.995	negative	2.816	<0.001	0.039	-0.546	-1.680	-0.012	<0.001	27.2	-0.593	-1.750	-0.021	<0.001	27.2	-0.423	-1.156	-0.005	<0.001	11.4
401.2358	positive	2.313	<0.001	0.001	-1.728	-4.168	-0.107	<0.001	**78.8**	-1.740	-4.230	-0.090	<0.001	**83.0**	-2.321	-3.501	-0.564	<0.001	**41.6**
317.1149	positive	2.205	<0.001	0.007	-0.560	-2.033	-0.016	<0.001	25.6	-0.516	-2.041	-0.003	<0.001	27.8	-1.285	-2.397	-0.050	<0.001	5.0
115.0757	positive	2.033	<0.001	0.007	1.063	0.003	2.821	<0.001	**38.4**	1.041	0.022	2.405	<0.001	37.6	1.898	0.224	2.801	<0.001	11.4
251.1408	positive	2.011	<0.001	0.007	-0.472	-1.246	-0.018	<0.001	21.6	-0.560	-1.461	-0.016	<0.001	23.2	-1.114	-2.098	-0.255	<0.001	3.4
166.0258	positive	1.956	<0.001	0.044	0.585	0.008	1.837	<0.001	29.4	0.667	0.015	1.847	<0.001	31.4	0.909	0.216	1.824	<0.001	4.0
73.0649	positive	1.864	<0.001	0.007	0.580	0.017	2.019	<0.001	33.0	0.585	0.016	2.031	<0.001	36.6	1.853	0.631	3.015	<0.001	9.6
400.2321	positive	1.823	<0.001	0.007	-2.367	-4.784	-0.113	<0.001	20.8	-2.375	-5.729	-0.069	<0.001	18.6	-3.281	-3.898	-2.128	<0.001	12.0
315.0416	negative	1.661	<0.001	0.011	-1.207	-3.427	-0.047	<0.001	**43.6**	-1.121	-3.393	-0.030	<0.001	**43.6**	-1.639	-2.560	-0.202	<0.001	8.4
267.1968	negative	1.647	<0.001	0.007	-0.392	-1.268	-0.018	<0.001	13.2	-0.485	-1.339	-0.013	<0.001	12.6	-0.932	-2.083	-0.059	<0.001	3.0
202.0685	positive	1.580	<0.001	0.033	-0.527	-1.867	-0.011	<0.001	18.4	-0.583	-1.785	-0.046	<0.001	16.4	-1.383	-2.337	-0.443	<0.001	1.2
343.1228	positive	1.468	<0.001	0.018	0.530	0.009	1.752	<0.001	24.6	0.432	0.013	1.199	<0.001	21.2	1.489	0.400	3.165	<0.001	2.4

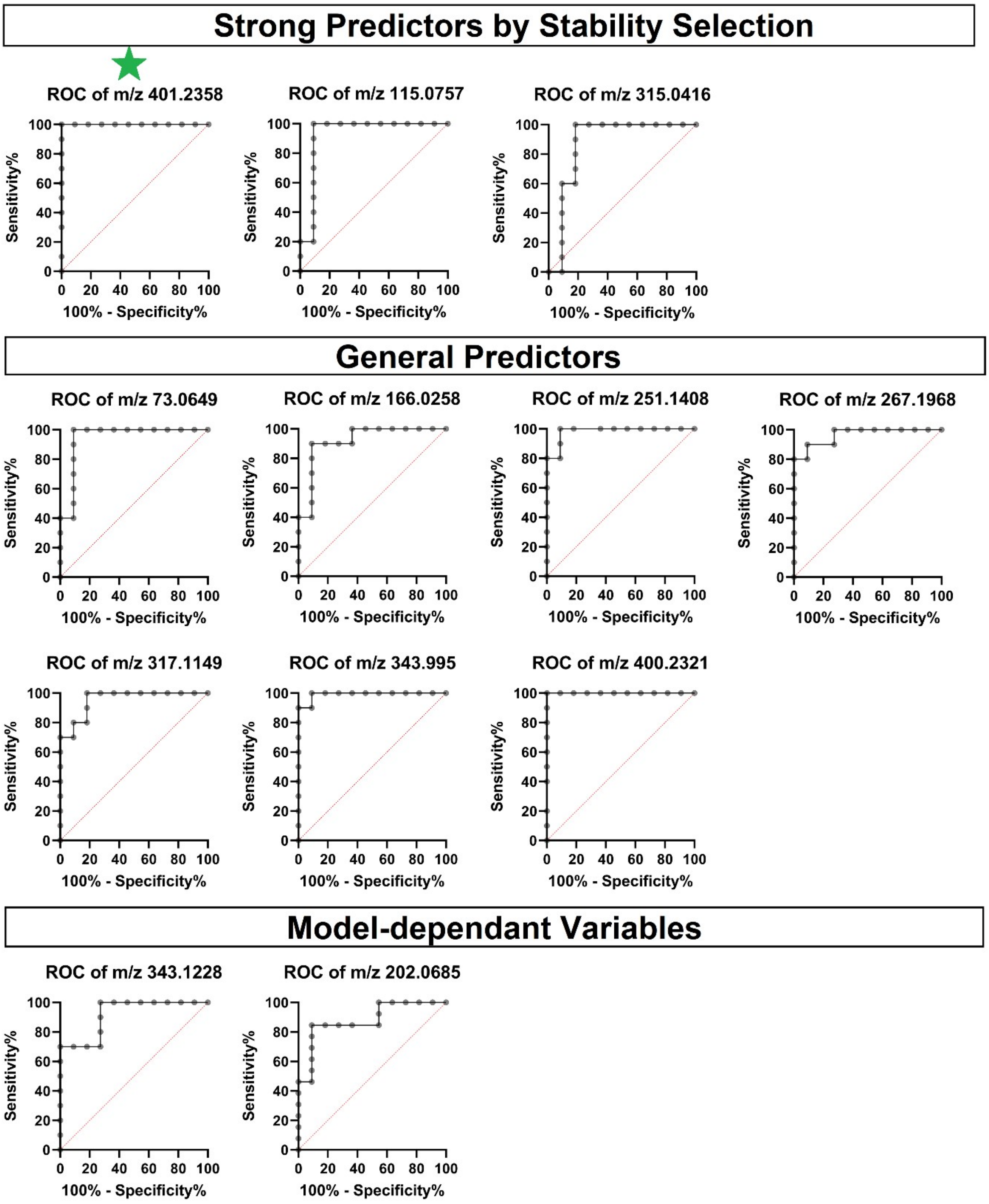

Fig. S9 Receiver Operating Characteristic (ROC) Curves for the discriminative ions identified from the conventional OPLS-DA based statistical workflow. The green star sign indicates the strongest candidate for a lameness indicator (m/z 401.2358, PG 35:4) discovered by stability selection. Relatively low specificity is seen in the model-dependant variables.
